# Clinical phenotypes of older adults with non-valvular atrial fibrillation not treated with oral anticoagulants by hierarchical cluster analysis in the ANAFIE Registry

**DOI:** 10.1371/journal.pone.0280753

**Published:** 2023-02-08

**Authors:** Shinya Suzuki, Takeshi Yamashita, Masaharu Akao, Hirotsugu Atarashi, Takanori Ikeda, Ken Okumura, Yukihiro Koretsune, Wataru Shimizu, Hiroyuki Tsutsui, Kazunori Toyoda, Atsushi Hirayama, Masahiro Yasaka, Takenori Yamaguchi, Satoshi Teramukai, Tetsuya Kimura, Yoshinori Morishima, Atsushi Takita, Hiroshi Inoue

**Affiliations:** 1 The Cardiovascular Institute, Tokyo, Japan; 2 Department of Cardiology, National Hospital Organization Kyoto Medical Center, Kyoto, Japan; 3 AOI Hachioji Hospital, Tokyo, Japan; 4 Department of Cardiovascular Medicine, Toho University Faculty of Medicine, Tokyo, Japan; 5 Division of Cardiology, Saiseikai Kumamoto Hospital Cardiovascular Center, Kumamoto, Japan; 6 National Hospital Organization Osaka National Hospital, Osaka, Japan; 7 Department of Cardiovascular Medicine, Graduate School of Medicine, Nippon Medical School, Tokyo, Japan; 8 Department of Cardiovascular Medicine, Faculty of Medical Sciences, Kyushu University, Fukuoka, Japan; 9 Department of Cerebrovascular Medicine, National Cerebral and Cardiovascular Center, Osaka, Japan; 10 Osaka Police Hospital, Osaka, Japan; 11 Department of Cerebrovascular Medicine and Neurology, Cerebrovascular Center, National Hospital Organization Kyushu Medical Center, Fukuoka, Japan; 12 Department of Biostatistics, Graduate School of Medical Science, Kyoto Prefectural University of Medicine, Kyoto, Japan; 13 Primary Medical Science Department, Daiichi Sankyo, Tokyo, Japan; 14 Data Intelligence Department, Daiichi Sankyo Co., Ltd., Tokyo, Japan; 15 Saiseikai Toyama Hospital, Toyama, Japan; Policlinico Casilino, ITALY

## Abstract

**Background:**

Although anticoagulants are indicated for many elderly patients with non-valvular atrial fibrillation (NVAF), some patients do not receive anticoagulant therapy, whose characteristics and outcomes are diverse.

**Methods and results:**

In this sub-analysis of the All Nippon AF In the Elderly (ANAFIE) Registry, the phenotypes of patients who were not receiving anticoagulants at baseline were evaluated by cluster analysis using Ward’s linkage hierarchical algorithm. Of 32,275 enrolled patients, 2445 (7.6%) were not receiving anticoagulants. Two clusters were identified: (1) elderly paroxysmal AF (PAF) patients with a high proportion of catheter ablation history (57%) and (2) very elderly patients with a high prevalence of previous major bleeding (43%). Respective mean ages were 80.9 and 84.2 years, mean CHA_2_DS_2_-VASc scores were 3.8 and 4.9, PAF prevalences were 100.0% and 31.4%, proportions of patients with catheter ablation history were 21.0% and 7.9%, and proportions of patients with a history of major bleeding were 4.0% and 10.8%. Annual incidence rates were 2.72% and 8.81% for all-cause death, 1.66% and 5.85% for major adverse cardiovascular or neurological events, 1.08% and 3.30% for stroke or systemic embolism, and 0.69% and 1.19% for major bleeding, respectively.

**Conclusions:**

In this cohort of elderly NVAF patients from the ANAFIE Registry who were not receiving anticoagulants, over half had PAF with a high proportion of catheter ablation history and a low incidence of adverse outcomes; for them, non-prescription of anticoagulants may be partially understandable, but they should be carefully monitored regarding AF burden or atrial cardiomyopathy and be adequately anticoagulated when adverse findings are detected. The remaining were very elderly patients with a high prevalence of previous major bleeding and a high incidence of adverse outcomes; for them, non-prescription of anticoagulants is inappropriate because of the high thromboembolic risk.

**Trial registration:**

**Registration:** http://www.umin.ac.jp/; Unique identifier: UMIN000024006.

## Introduction

Atrial fibrillation (AF) is one of the most common arrhythmias, and it is associated with a substantially increased risk of mortality and morbidity, especially ischemic stroke and heart failure [1]. Advancing age is a critical risk factor for AF, and because of population aging, the incidence and prevalence of AF are currently increasing worldwide [2]. Anticoagulation therapy can reduce the risk of ischemic stroke in AF patients by two-thirds but is associated with an increased risk of bleeding [3]. Of note, advanced age increases the risk of both ischemic stroke and bleeding in patients receiving anticoagulation therapy [4, 5], with the increased bleeding risk being one of the reasons for the underuse of anticoagulation therapy in elderly patients [6].

Before the introduction of direct oral anticoagulants (DOACs), the prescription rate of anticoagulants for patients with AF was approximately 50% in a Japanese clinical practice registry (Fushimi-AF) [6]. In a pooled analysis of three AF registries in Japan (Shinken, J-RHYTHM and Fushimi-AF), the incidence of ischemic stroke in patients not treated with anticoagulants despite having a high thromboembolic risk (CHADS_2_ score ≥2) was 2.47% per year [7]. In the main analysis of the nationwide All Nippon AF In the Elderly (ANAFIE) Registry, which enrolled elderly (age ≥75 years) patients with AF (n = 32,275), the anticoagulant prescription rate was 92.4% [8], which was remarkably higher than that in the pre-DOAC era [6]. In this registry, the incidence of ischemic stroke in elderly patients with AF was 1.74%, 1.50%, and 1.09% per year in patients who received no anticoagulation therapy, warfarin, or DOACs, respectively [8].

Although a high anticoagulant prescription rate was reported in the ANAFIE Registry, it is unclear whether this should be further improved. The decision to withhold anticoagulants from elderly patients with AF who potentially have a high risk of thromboembolism should be based on robust clinical grounds. One reason not to prescribe anticoagulants would be the physician’s concern about higher bleeding risk, irrespective of the thromboembolic risk [9]. Another reason may be the lower risk of AF-associated thromboembolism compared with bleeding risk [10]. Thus, it is likely that there may be two phenotypes of elderly patients with AF who are not treated with anticoagulation therapy: those with a very low thromboembolic risk and those with a very high bleeding risk.

Several cluster analyses have reportedly identified up to seven clinical phenotypes in patients with AF, with many of these analyses indicating that rates of mortality or major adverse cardiovascular or neurological events (MACNE) could be stratified by cluster [11–16]. For example, in an analysis of patients enrolled in the ORBIT-AF Registry, the incidence rates of MACNE were 2.58%, 3.97%, 5.10%, and 6.12% per year for clusters defined by low comorbidity rates, younger age or comorbid behavioral disorders, device implantation, or atherosclerotic comorbidities, respectively [11].

To our knowledge, no cluster analyses have been performed in elderly patients with AF who were not receiving anticoagulation therapy. Therefore, we performed a cluster analysis for patients enrolled in the ANAFIE Registry who were not receiving treatment with anticoagulants at baseline to identify a better clinical management for this population.

## Methods

### Study design and participants

The ANAFIE Registry (UMIN Clinical Trials Registry UMIN000024006) was a multicenter, prospective, observational study of elderly (age ≥75 years) Japanese patients with non-valvular AF (NVAF). The registry was conducted in accordance with the Declaration of Helsinki and local regulatory requirements and ethical guidelines for clinical studies in Japan. All participants provided written informed consent prior to enrollment. The study was approved by the Ethics Committees of The Cardiovascular Research Institute (Tokyo, Japan) and is registered with the University hospital Medical Information Network with the identifier UMIN000024006. The study began recruitment in October 2016, and participants were followed up for a minimum of 2 years. Details of the study design and eligibility criteria have been published previously [17].

### Outcomes

The outcomes of interest in this analysis were stroke or systemic embolic events (SEE), major bleeding (as defined by the International Society on Thrombosis and Haemostasis), intracranial hemorrhage (ICH), all bleeding, death from cardiovascular disease, all-cause death, heart failure requiring hospitalization, and MACNE. MACNE is a composite of cardiovascular death, stroke, SEE, and myocardial infarction [11, 13]. In this analysis, the lack of data precluded the inclusion of transient ischemic attack (TIA) in MACNE.

### Patient categorization

The study participants were categorized via the following steps.

**1) Data preparation**

For this analysis, predicted rather than measured values were used for continuous variables to avoid the impact of outliers and missing data (data were missing for the following continuous variables: body mass index [14.1%], systolic blood pressure [10.3%], hemoglobin [18.7%], glycated hemoglobin [48.4%], estimated creatinine clearance [CrCl, 24.5%], and number of medications [4.6%]). Multiple regression models were developed using age, sex, and other categorical variables, and values for each continuous variable were predicted. For the proportion of categorical variables (no missing data, except for patients with a fall within 1 year before enrollment [missing: 11.3%]), predictive probabilities were calculated by multivariate logistic regression analysis using all categorical variables (other than the predicted variable) and age. These were used in the cluster analysis as an alternative to the categorical variable, providing a gradual distribution instead of a dichotomized category. The predicted value of a continuous variable can be interpreted to represent the patient characteristics that are related to the variable rather than the value itself.

**2) Hierarchical cluster analysis**

A hierarchical cluster analysis was performed using Ward’s linkage hierarchical algorithm on predicted values for continuous variables and predictive probabilities for categorical variables [11].

### Statistical methods

Baseline characteristics are described using summary statistics, with mean ± standard deviation for continuous variables and n (%) for categorical variables. The risk of ischemic stroke was determined for each patient at baseline using the CHADS_2_ score (congestive heart failure, hypertension, age ≥75 years, diabetes mellitus, and prior stroke, TIA, or thromboembolism), the CHA_2_DS_2_-VASc score (congestive heart failure, hypertension, age ≥75 years, diabetes mellitus, prior stroke, TIA, or thromboembolism, vascular disease, age 65–74 years, and sex category), and the HELT-E_2_S_2_ score (hypertension, elderly [75–84 years], low body mass index, type of AF, extremely elderly [≥85 years], and previous stroke) [18]. The risk of major bleeding was determined for each patient at baseline using the HAS-BLED score (hypertension, abnormal renal and liver function, stroke, bleeding, labile international normalized ratio, elderly, and drugs or alcohol). Statistical differences between the two clusters were tested using the unpaired t-test for continuous variables and Fisher’s exact test for categorical variables. The cumulative incidences of stroke/SEE, all bleeding, major bleeding, ICH, cardiovascular death, all-cause death, and MACNE during the 2-year follow-up were depicted using the Kaplan–Meier method, and the statistical differences between the two clusters were tested using the log-rank test. All statistical analyses were conducted using SPSS version 28.0 (IBM Corp., Armonk, NY, USA) and STATA 13.0 (STATA Corp., College Station, Texas, USA).

## Results

### Patients and clusters

**Fig 1:**
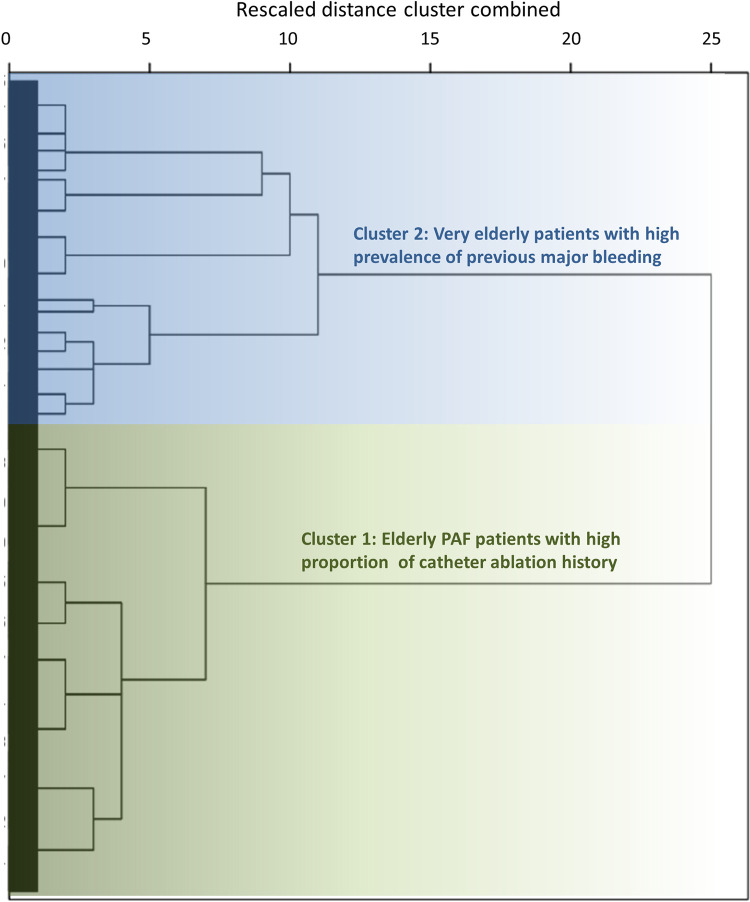
Dendrogram of the hierarchical cluster analysis. Abbreviation: PAF, paroxysmal atrial fibrillation.

Among the 32,275 patients enrolled in the ANAFIE Registry, 2445 (7.6%) patients who were not receiving anticoagulation therapy at baseline were included in this analysis. The baseline characteristics of this subgroup of patients are summarized in **Table 1**. The average age was 82.3 years, and 1273 (52.1%) were male. Two clusters of patients were identified (**Fig 1**): (1) elderly patients with paroxysmal AF (PAF) and a high proportion of catheter ablation history; and (2) very elderly patients with a high prevalence of previous major bleeding.

**Table 1:** Patient characteristics.

	Total	Elderly PAF with	Very elderly with	p value
		high prevalence of	high prevalence of	
		catheter ablation	bleeding history	
		history		
	n = 2445	n = 1388	n = 1057	
Men	1273 (52.1)	709 (51.1)	564 (53.4)	0.270
Age, years	82.3 ± 5.5	80.9 ± 4.8	84.2 ± 5.8	<0.001
75 to <80	893 (36.5)	631 (45.5)	262 (24.8)	<0.001
80 to <85	725 (29.7)	422 (30.4)	303 (28.7)	
85 to <90	547 (22.4)	256 (18.4)	291 (27.5)	
90 to <95	224 (9.2)	71 (5.1)	153 (14.5)	
95 to <100	51 (2.1)	8 (0.6)	43 (4.1)	
> = 100	5 (0.2)	0 (0)	5 (0.5)	
> = 85	827 (33.8)	335 (24.1)	492 (46.5)	<0.001
BMI, kg/m^2^	22.7 ± 3.5	22.7 ± 3.4	22.6 ± 3.7	0.674
SBP, mmHg	130 ± 17.5	131.6 ± 16.4	127.9 ± 18.6	<0.001
DBP, mmHg	70.4 ± 11.5	70.9 ± 10.8	69.8 ± 12.3	0.037
HbA1c, %	6 ± 0.8	5.9 ± 0.6	6.2 ± 1	<0.001
Creatinine clearance, mL/min	45.6 ± 18.2	49.8 ± 16.9	40.4 ± 18.5	<0.001
<15 or dialysis	64 (2.6)	15 (1.1)	49 (4.6)	<0.001
>15 to <30	307 (12.6)	97 (7)	210 (19.9)	
>30 to <50	737 (30.1)	402 (29)	335 (31.7)	
>50 to <80	675 (27.6)	466 (33.6)	209 (19.8)	
>80	64 (2.6)	41 (3)	23 (2.2)	
CHADS_2_ score	2.6 ± 1.2	2.3 ± 0.9	3.2 ± 1.2	<0.001
CHA_2_DS_2_-VASc score	4.3 ± 1.4	3.8 ± 1.1	4.9 ± 1.5	<0.001
HELT-E_2_S_2_ score	3.0 ± 1.3	2.4 ± 1.0	3.8 ± 1.2	<0.001
HAS-BLED score	2.0 ± 0.9	1.7 ± 0.8	2.2 ± 1.0	<0.001
History of major bleeding	170 (7.0)	56 (4.0)	114 (10.8)	<0.001
AF type				<0.001
Paroxysmal	1720 (70.3)	1388 (100)	332 (31.4)	
Persistent/long persistent	255 (10.4)	0 (0.0)	255 (24.1)	
Permanent	180 (7.4)	0 (0.0)	180 (17.0)	
Unknown	290 (11.9)	0 (0.0)	290 (27.4)	
Non-pharmacological AF therapy	580 (23.7)	390 (28.1)	190 (18)	<0.001
Catheter ablation	376 (15.4)	292 (21.0)	84 (7.9)	<0.001
Electrical defibrillation	40 (1.6)	22 (1.6)	18 (1.7)	0.872
ICD	17 (0.7)	10 (0.7)	7 (0.7)	0.999
Pacemaker	189 (7.7)	93 (6.7)	96 (9.1)	0.032
Others	13 (0.5)	5 (0.4)	8 (0.8)	0.261
Comorbidities				
Heart failure	752 (30.8)	238 (17.1)	514 (48.6)	<0.001
Myocardial infarction	138 (5.6)	28 (2.0)	110 (10.4)	<0.001
Hypertension	1821 (74.5)	1015 (73.1)	806 (76.3)	0.083
Diabetes mellitus	580 (23.7)	201 (14.5)	379 (35.9)	<0.001
Chronic kidney disease	441 (18.0)	179 (12.9)	262 (24.8)	<0.001
Dyslipidemia	1066 (43.6)	598 (43.1)	468 (44.3)	0.564
Cerebrovascular disease	468 (19.1)	153 (11.0)	315 (29.8)	<0.001
Gastrointestinal disease	805 (32.9)	412 (29.7)	393 (37.2)	<0.001
Severe liver disease	22 (0.9)	14 (1.0)	8 (0.8)	0.666
Active cancer	284 (11.6)	164 (11.8)	120 (11.4)	0.750
Dementia	248 (10.1)	80 (5.8)	168 (15.9)	<0.001
Fall within 1 year	167 (6.8)	67 (4.8)	100 (9.5)	<0.001
Medication				
Antiarrhythmic drugs for AF rhythm control	646 (26.4)	488 (35.2)	158 (14.9)	<0.001
Antiarrhythmic drugs for AF rate control	714 (29.2)	324 (23.3)	390 (36.9)	<0.001
Antiplatelet	840 (34.4)	386 (27.8)	454 (43.0)	<0.001
Proton pump inhibitor	780 (31.9)	375 (27.0)	405 (38.3)	<0.001
P-glycoprotein inhibitor	48 (2.0)	20 (1.4)	28 (2.6)	0.038
Number of medications	5.9 ± 3.3	5.3 ± 3.1	6.6 ± 3.4	<0.001

Data are presented as n (%) or mean ± standard deviation.

Abbreviations: AF, atrial fibrillation; BMI, body mass index; DBP, diastolic blood pressure; ICD, implantable cardioverter-defibrillator; SBP, systolic blood pressure.

#### Cluster 1: Elderly patients with PAF and a high proportion of catheter ablation history (n = 1388, 57%)

Cluster 1 accounted for more than half of the study population. The average age was 80.9 years, and 51.1% were male. PAF accounted for 100.0% of patients in this cluster, and 21.0% had undergone catheter ablation at baseline. Regarding thromboembolic risk, mean scores were 2.3 for CHADS_2_, 3.8 for CHA_2_DS_2_-VASc, and 2.4 for HELT-E_2_S_2_. The prevalence of comorbidities was relatively low and included heart failure (17.1%), myocardial infarction (2.0%), cerebrovascular disease (11.0%), diabetes mellitus (14.5%), and history of major bleeding (4.0%). Antiplatelets were prescribed for 27.8% of patients, and the average number of medications prescribed was 5.3.

#### Cluster 2: Very elderly patients with a high prevalence of previous major bleeding (n = 1057, 43%)

This cluster accounted for less than half of the study population. The average age was 84.2 years, and 53.4% were male. PAF accounted for 31.4% of these patients, and 7.9% had undergone catheter ablation at baseline. Regarding thromboembolic risk, mean risk scores (3.2 for CHADS_2_, 4.9 for CHA_2_DS_2_-VASc, and 3.8 for HELT-E_2_S_2_ score) were higher in cluster 2 compared with cluster 1. The prevalence of comorbidities was higher than in cluster 1 and included heart failure (48.6%), myocardial infarction (10.4%), cerebrovascular disease (29.8%), diabetes mellitus (35.9%), and history of major bleeding (10.8%). Antiplatelets were prescribed for 43.0% of these patients, and the average number of medications prescribed was 6.6.

### Outcomes

**Fig 2:**
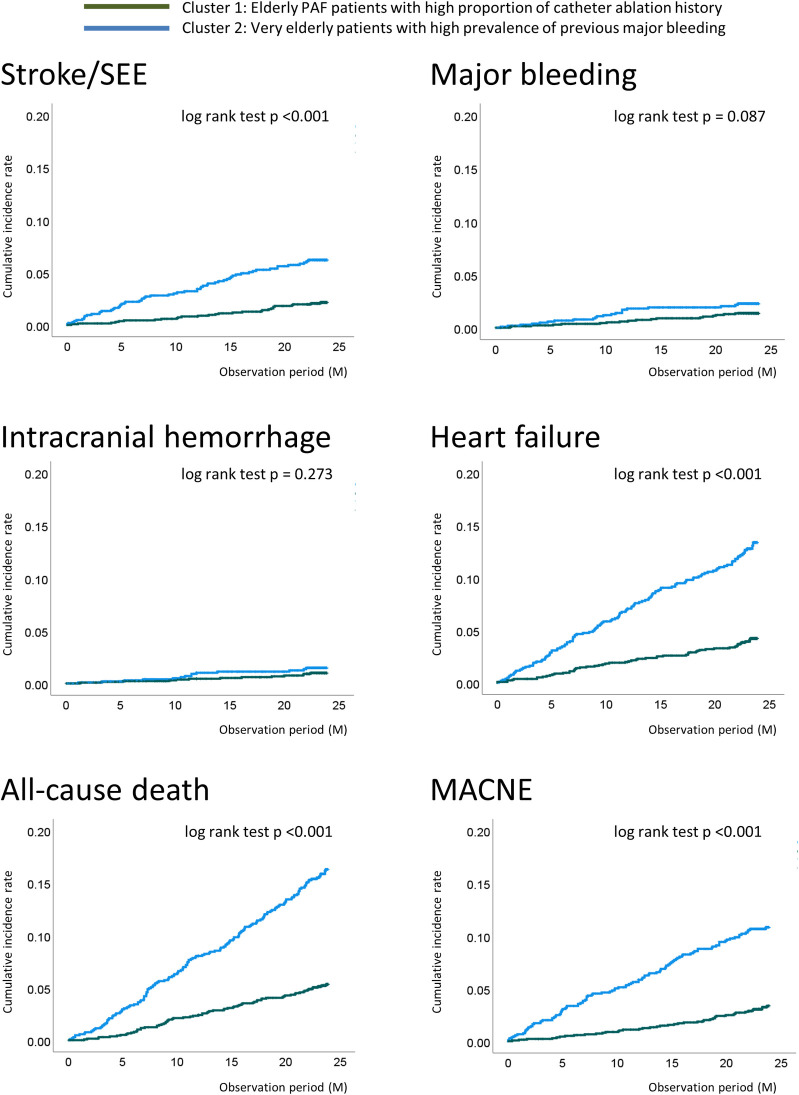
Kaplan–Meier curves for stroke/SEE, all bleeding, major bleeding, intracranial hemorrhage, all-cause death, and major adverse cardiovascular or neurological event. Abbreviations: PAF, paroxysmal atrial fibrillation; SEE, systemic embolic events; MACNE, major adverse cardiovascular or neurological events.

Annual incidence rates for clusters 1 and 2 were 2.72% and 8.81% for all-cause death, 1.66% and 5.85% for MACNE, 1.08% and 3.30% for stroke/SEE, 0.69% and 1.19% for major bleeding, and 2.14% and 7.14% for heart failure requiring hospitalization, respectively (**Table 2**). Kaplan–Meier curves for outcome events are shown in **Fig 2**. For all outcomes analyzed other than bleeding events, incidence rates for cluster 2 were significantly higher than those for cluster 1 (log-rank test, p <0.001). Incidence rates of major bleeding and ICH were numerically higher in cluster 2 than in cluster 1, but the difference did not reach statistical significance (p = 0.087 and 0.273, respectively).

**Table 2:** Annualized incidence rate for clinical outcomes by clusters.

	Total	Elderly PAF with high prevalence of catheter ablation history	Very elderly with high prevalence of bleeding history
	n = 2445	n = 1388	n = 1057
Stroke/SEE	2.00 (1.58, 2.41)	1.08 (0.68, 1.48)	3.30 (2.47, 4.14)
Ischemic stroke	1.74 (1.35, 2.13)	0.93 (0.56, 1.30)	2.91 (2.13, 3.69)
Hemorrhagic stroke	0.22 (0.09, 0.36)	0.19 (0.02, 0.36)	0.27 (0.03, 0.50)
SEE	0.04 (0.00, 0.11)	0.00 (0.00, 0.00)	0.11 (0.00, 0.26)
All bleeding	2.65 (2.17, 3.14)	2.31 (1.72, 2.89)	3.14 (2.33, 3.96)
Major bleeding	0.90 (0.62, 1.18)	0.69 (0.37, 1.01)	1.19 (0.69, 1.69)
ICH	0.61 (0.38, 0.83)	0.50 (0.23, 0.77)	0.75 (0.36, 1.15)
GI bleeding	1.34 (1.00, 1.68)	1.24 (0.81, 1.67)	1.47 (0.92, 2.02)
All-cause death	5.26 (4.58, 5.93)	2.72 (2.09, 3.35)	8.81 (7.46, 10.16)
Cardiovascular death	1.63 (1.26, 2.01)	0.65 (0.34, 0.96)	3.01 (2.22, 3.80)
Heart failure admission	4.19 (3.58, 4.80)	2.14 (1.57, 2.70)	7.14 (5.90, 8.38)
MACNE	3.38 (2.84, 3.93)	1.66 (1.16, 2.16)	5.85 (4.73, 6.96)

Notes: Data in the table are the % per patient-year (95% confidence interval). a MACNE was defined as the composite of stroke/SEE, myocardial infarction, and cardiovascular death.

Abbreviations: GI = gastrointestinal; ICH = intracranial hemorrhage; SEE = systemic embolic events

MACNE = major adverse cardiovascular or neurological event

## Discussion

### Main findings

In this analysis from the ANAFIE Registry, two distinct clinical phenotypes were identified in a subgroup of patients not receiving anticoagulation therapy at baseline by hierarchical cluster analysis: (1) elderly patients with PAF and a high proportion of catheter ablation history (57%), and (2) very elderly patients with a high prevalence of previous major bleeding (43%). Cluster 1 was characterized by a high prevalence of PAF and a high proportion of patients who underwent catheter ablation at baseline. Patients in cluster 2 were older and had a high comorbidity burden, with a particularly high prevalence of heart failure (48.6%) and a history of major bleeding (10.8%). The incidence of adverse outcomes was significantly higher in cluster 2 than in cluster 1, except for bleeding events.

### Cluster 1: Elderly PAF with high proportion of catheter ablation history

This cluster accounted for more than half of the study population. In this cluster, PAF accounted for 100% of the patients, and catheter ablation had already been performed in approximately 20%. As a result, the AF burden seemed to be low, and this may be one of the reasons for the lack of anticoagulant use in this cluster. This speculation could be supported by the lower incidence rate of ischemic stroke (1.08% per year) in these patients, despite the lack of anticoagulation, which was almost identical to that in patients prescribed DOACs in the ANAFIE Registry (1.09%) [8]. A low AF burden, as determined by an implanted device, has been shown to be associated with a low incidence of stroke [10]. However, given that asymptomatic AF is not uncommon in older adults, in clinical practice it can be challenging to identify a low AF burden by symptoms alone [19]. For this cluster, the physicians might have appropriately determined the AF burden as low and consequently selected no anticoagulation therapy, but it is unknown how the AF burden was estimated.

Based on the mean values of conventional risk scores for thromboembolic risk, such as CHADS_2_ and CHA_2_DS_2_-VASc scores of 2.3 and 3.8, respectively, anticoagulation would have been strongly recommended for patients in this cluster. Interestingly, the recently developed risk score of thromboembolic risk, HELT-E_2_S_2_ score, indicated modest risk (mean, 2.4) in this cluster. In the J-RISK study [18], the incidence of ischemic stroke was 2.8%/year for CHADS_2_ ≥2 and 2.3%/year for CHA_2_DS_2_-VASc ≥2, but 1.4%/year for a HELT-E_2_S_2_ score of 2 points. Based on the risk assessment for ischemic stroke by HELT- E_2_S_2_ score, in which 2 points are assigned for age ≥85 years and 1 point for non-PAF, the lack of oral anticoagulant (OAC) use for this cluster may be partially understandable. However, it is essential that elderly AF patients be carefully monitored regarding AF burden or atrial cardiomyopathy, and they should be adequately anticoagulated when adverse findings are detected.

### Cluster 2: Very elderly with a high prevalence of previous major bleeding

This cluster accounted for nearly half of the elderly patients not anticoagulated in the ANAFIE Registry. These patients had a higher mean CHADS_2_ score (3.2), CHA_2_DS_2_-VASc score (4.9), and HELT-E_2_S_2_ score (3.8), and higher annual incidence of ischemic stroke (2.91%). Thromboembolic risks for patients in this cluster appeared to be similar to those observed in other Japanese cohorts with similar thromboembolic risk scores. For example, in a pooled analysis of three Japanese registries, the annual incidence rate of ischemic stroke in patients with AF not receiving anticoagulation therapy was 2.66% for those with a CHADS_2_ score of 3 and 4.43% for those with a CHA_2_DS_2_-VASc score of 5 [7]. Similarly, in the J-RISK study, the annual incidence rate of ischemic stroke in patients with AF and a HELT-E_2_S_2_ score of 4 and without anticoagulation therapy was 3.96% [18].

Despite having a mean HAS-BLED score of <3 (i.e., not considered to be at high risk of bleeding), the patients in this cluster did have high bleeding risk characteristics, such as advanced age (mean 84.2 years), mean CrCl of 40.4 mL/min, and a relatively high prevalence of previous major bleeding (10.8%) and dementia (15.9%). These characteristics are similar to those reported in the ELDERCARE-AF trial [9], which recruited very elderly Japanese patients with high thromboembolic and bleeding risks who were deemed inappropriate candidates for standard-dose anticoagulation by physicians. In that trial, the mean age was 86.6 years, 22.6% had a history of major bleeding, 16.3% had dementia, and the mean CrCl was 36.3 mL/min. The participants also had a mean HAS-BLED score comparable to patients in cluster 2 (2.3 vs 2.2). In the placebo arm of ELDERCARE-AF, the annual incidence rates of major bleeding and ICH were 1.8% and 0.6%, respectively, which were comparable to those in cluster 2 (1.19% and 0.75%, respectively), although the incidence of all bleeding was higher in ELDERCARE-AF compared with this cluster (45.0% vs 3.14%). Although the annual incidence rate of stroke or SEE in cluster 2 (stroke or SEE: 3.30%; ischemic stroke: 2.91%) was lower than that in the placebo arm of ELDERCARE-AF (stroke or SEE: 6.7%; ischemic stroke: 5.9%), the incidence rate was three times higher than that in cluster 1. Therefore, non-prescription of OACs is inappropriate in this cluster because of the high thromboembolic risk.

It seems likely that the decision not to prescribe anticoagulants for patients in this cluster was driven by a high bleeding risk irrespective of thromboembolic risk. In the ELDERCARE-AF trial, very low-dose edoxaban (15 mg) was superior to placebo in preventing stroke or SEE and did not result in a significantly higher incidence of major bleeding than placebo [9]. Of note, very low-dose edoxaban did not increase the risk of life-threatening bleeding, including ICH, but significantly increased gastrointestinal bleeding risk [9]. Given the very similar profiles of patients in this cluster compared with those enrolled in ELDERCARE-AF, very low-dose anticoagulation, such as edoxaban 15 mg, could be carefully considered for the cohort of very elderly patients with a significant bleeding history that was identified in this analysis. Moreover, in such high-risk AF patients, left atrial appendage closure may also be considered for effective stroke prevention with minimized bleeding events [20].

### Comparison to previous cluster analyses

Previous Japanese analyses in patients with AF have identified clusters of patients with PAF that show some similarities to cluster 1 in this analysis. For example, an analysis of the KiCS-AF Registry identified a cluster of younger patients with PAF that accounted for almost half (48.4%) of the total patients enrolled in the registry [11]. Patients in this cluster had a mean age of 65.9 years, and almost all had PAF (96.5%). The cumulative incidence of MACNE at 1 year for this cluster was approximately 2% based on Kaplan–Meier analysis. Similarly, a cluster of younger patients (4.9% of all patients) was identified in the Fushimi-AF Registry [13]. Patients in this cluster had a mean age of 48.3 years, and 76.6% had PAF. The incidence of MACNE in this cluster was only 0.5% per year. Cluster 1 in the present analysis had a mean age of 80.9 years and the prevalence of PAF was 100.0%. Despite their higher age, the incidence of MACNE in this cluster was 1.66% per year, which is similar to that in the younger paroxysmal AF cluster in the KiCS-AF Registry [11] but higher than that in the younger age cluster in the Fushimi-AF Registry [13].

Similar to cluster 2, a very elderly patient cluster was also identified in the Fushimi-AF Registry (6.1% of the total patients) [13]. Patients in this cluster had a similar mean age to those in cluster 2 (83.4 vs 84.2 years), but the proportion of patients with a history of major bleeding was considerably lower (1.9% vs 10.8%). The prescription rate for OACs was 48.5% in the very elderly cluster in Fushimi-AF compared with 0% in cluster 2. The annual incidence rates for all-cause death and major bleeding were higher in this cluster in Fushimi-AF compared with cluster 2 (all-cause death: 15.9% vs 8.81%; major bleeding 3.8% vs 1.19%). Lower annual incidence rates were also reported for cluster 2 in this analysis vs the very elderly cluster in Fushimi-AF for MACNE (5.85% vs 11.35%) and stroke/SEE (3.30% vs 7.04%).

### Limitations

Some limitations of this study warrant mention, including those inherent to the observational study design. The incidence of major bleeding in the overall ANAFIE Registry was lower than expected, which may have also affected the results of this sub-analysis. In addition, data were missing for some continuous variables; however, these were replaced with predicted values. Finally, our findings are limited to Japanese patients eligible for outpatient management and cannot be generalized to other ethnic populations.

## Conclusions

Elderly patients with NVAF who were not receiving OACs at baseline in the ANAFIE Registry were phenotypically heterogeneous. Two clusters of patients were identified; one had a low incidence of adverse outcomes during follow-up and primarily comprised patients with PAF. This cluster accounted for over half of the overall population of unanticoagulated patients in ANAFIE. For this cluster, non-prescription of anticoagulants may be partially understandable, but they should be carefully monitored regarding AF burden or atrial cardiomyopathy and be adequately anticoagulated when adverse findings are detected. The other cluster comprised the remaining patients (i.e., very elderly patients with a high prevalence of major bleeding) and had a high incidence of adverse outcomes. For this cluster, non-prescription of anticoagulants is inappropriate because of the high thromboembolic risk.

## Supporting information

S1 Checklist Author formatting checklist. (DOCX)

S1 File ANAFIE code explanation 1. (PDF)

S2 File ANAFIE code explanation 2. (PDF)

S3 File Analysis code 1. (PDF)

S4 File Analysis code 2. (PDF)

S5 File Analysis code 3. (PDF)

S6 File Analysis code 4. (PDF)

S7 File Analysis code 5. (PDF)

## Abbreviations and Acronyms

AF: atrial fibrillation
ANAFIE: All Nippon AF In the Elderly
CrCl: creatinine clearance
DOAC: direct oral anticoagulant
ICH: intracranial hemorrhage
MACNE: mortality or major adverse cardiovascular or neurological event
NVAF: non-valvular atrial fibrillation
OAC: oral anticoagulant
PAF: paroxysmal atrial fibrillation
SEE: systemic embolic events
TIA: transient ischemic attack
