# Supplementary material for: Clinical phenotypes of older adults with non-valvular atrial fibrillation not treated with oral anticoagulants by hierarchical cluster analysis in the ANAFIE Registry
Source: PLoS One. 2023 Feb 8;18(2):e0280753. doi: 10.1371/journal.pone.0280753 (PMC9907799; doi:10.1371/journal.pone.0280753)
Supplement: S2 File — (PDF) [file pone.0280753.s003.pdf]

ADTTE_info

PARAM	PARAMN	TIME_M
Stroke/Systemic Embolism	101	TIME / 365.25 * 12
Stroke	102	TIME / 365.25 * 12
Ischemic Stroke	103	TIME / 365.25 * 12
Hemorrhagic Stroke	104	TIME / 365.25 * 12
Systemic Embolism	105	TIME / 365.25 * 12
Major Bleeding	106	TIME / 365.25 * 12
Clinically important bleeding	107	TIME / 365.25 * 12
Minor bleeding	108	TIME / 365.25 * 12
All bleeding	109	TIME / 365.25 * 12
Intracranial bleeding	110	TIME / 365.25 * 12
Gastrointestinal Bleeding	111	TIME / 365.25 * 12
Heart Disease Events	112	TIME / 365.25 * 12
Ischemic Heart Disease	113	TIME / 365.25 * 12
Myocardial Infarction	114	TIME / 365.25 * 12
Cardiac Insufficiency Requiring Hospitalization	115	TIME / 365.25 * 12
Death, Sudden, Cardiac	116	TIME / 365.25 * 12
Cardiovascular Events	117	TIME / 365.25 * 12
Death from cardiovascular disease	118	TIME / 365.25 * 12
All cause mortality	119	TIME / 365.25 * 12
Fractures and falls	120	TIME / 365.25 * 12
Fractures	121	TIME / 365.25 * 12
Falls	122	TIME / 365.25 * 12
Net Clinical Outcome	123	TIME / 365.25 * 12
