# Supplementary material for: Clinical phenotypes of older adults with non-valvular atrial fibrillation not treated with oral anticoagulants by hierarchical cluster analysis in the ANAFIE Registry
Source: PLoS One. 2023 Feb 8;18(2):e0280753. doi: 10.1371/journal.pone.0280753 (PMC9907799; doi:10.1371/journal.pone.0280753)
Supplement: S3 File — (PDF) [file pone.0280753.s004.pdf]

*Description in manuscript:
"1) Data preparation
For this analysis, predicted rather than measured values were used for continuous variables to avoid the impact of outliers and missing data (data were missing for the following continuous variables: body mass index [14.1%], systolic blood pressure [10.3%], hemoglobin [18.7%], glycated hemoglobin [48.4%], estimated creatinine clearance [CrCl, 24.5%], and number of medications [4.6%]). Multiple regression models were developed using age, sex, and other categorical variables, and values for each continuous variable were predicted."

* Code to prepare the predicted continuous variables

```
COMPUTE AD_SELECTED = 0.
EXECUTE.

IF(AD_FLAG = 0)    AD_SELECTED = 1.
EXECUTE.

COMPUTE ADYN_NO = 0.
EXECUTE.

IF(ADYN = 0)    ADYN_NO = 1.
EXECUTE.

COMPUTE AFTYP_dammy1 = 0.
EXECUTE.

IF(AFTYP = 1)    AFTYP_dammy1 = 1.
EXECUTE.

COMPUTE AFTYP_dammy2 = 0.
EXECUTE.

IF(AFTYP = 2)    AFTYP_dammy2 = 1.
EXECUTE.
```

```
COMPUTE AFTYP_dammy3 = 0.
EXECUTE.

IF(AFTYP = 3)    AFTYP_dammy3 = 1.
EXECUTE.

COMPUTE AFTYP_dammy4 = 0.
EXECUTE.

IF(AFTYP = 4)    AFTYP_dammy4 = 1.
EXECUTE.

COMPUTE TCMCNT_WA_CNT = TCMCNT_WA.
EXECUTE.

IF(TCMCNT_WA = 999)    TCMCNT_WA_CNT = ''.
EXECUTE.




USE ALL.
COMPUTE filter_$=(AD_SELECTED = 1 & ADYN_NO =1).
VARIABLE LABELS filter_$ 'AD_SELECTED = 1 & ADYN_NO =1 (FILTER)'.
VALUE LABELS filter_$ 0 'Not Selected' 1 'Selected'.
FORMATS filter_$ (f1.0).
FILTER BY filter_$.
EXECUTE.

REGRESSION
  /MISSING LISTWISE
  /STATISTICS COEFF OUTS CI(95) R ANOVA
  /CRITERIA=PIN(.05) POUT(.10)
  /NOORIGIN
```

```
   /DEPENDENT BMI_BL
   /METHOD=ENTER
SEX
AGE
CHFDFL
HTD1YN
DIAMFL
HYPURNYN
LMDYN
CVACYN
TEDYN
GASDYN
SLVDYN
MGTYN
BLDFL
ALZHYN
AFTHSPE1
ARTD1
ARTD2
APLD
PRPI
PGPI
AFTYP_dammy1
AFTYP_dammy2
AFTYP_dammy3
AFTYP_dammy4
   /SAVE PRED.

REGRESSION
   /MISSING LISTWISE
   /STATISTICS COEFF OUTS CI(95) R ANOVA
   /CRITERIA=PIN(.05) POUT(.10)
   /NOORIGIN
   /DEPENDENT SBP_BL
   /METHOD=ENTER
SEX
```

```
AGE
CHFDFL
HTD1YN
DIAMFL
HYPURNYN
LMDYN
CVACYN
TEDYN
GASDYN
SLVDYN
MGTYN
BLDFL
ALZHYN
AFTHSPE1
ARTD1
ARTD2
APLD
PRPI
PGPI
AFTYP_dammy1
AFTYP_dammy2
AFTYP_dammy3
AFTYP_dammy4
  /SAVE PRED.

REGRESSION
  /MISSING LISTWISE
  /STATISTICS COEFF OUTS CI(95) R ANOVA
  /CRITERIA=PIN(.05) POUT(.10)
  /NOORIGIN
  /DEPENDENT CRC
  /METHOD=ENTER
SEX
AGE
CHFDFL
HTD1YN
```

```
DIAMFL
HYPURNYN
LMDYN
CVACYN
TEDYN
GASDYN
SLVDYN
MGTYN
BLDFL
ALZHYN
AFTHSPE1
ARTD1
ARTD2
APLD
PRPI
PGPI
AFTYP_dammy1
AFTYP_dammy2
AFTYP_dammy3
AFTYP_dammy4
  /SAVE PRED.

REGRESSION
  /MISSING LISTWISE
  /STATISTICS COEFF OUTS CI(95) R ANOVA
  /CRITERIA=PIN(.05) POUT(.10)
  /NOORIGIN
  /DEPENDENT HEMO_VAL
  /METHOD=ENTER
SEX
AGE
CHFDFL
HTD1YN
DIAMFL
HYPURNYN
LMDYN
```

```
CVACYN
TEDYN
GASDYN
SLVDYN
MGTYN
BLDFL
ALZHYN
AFTHSPE1
ARTD1
ARTD2
APLD
PRPI
PGPI
AFTYP_dammy1
AFTYP_dammy2
AFTYP_dammy3
AFTYP_dammy4
  /SAVE PRED.

REGRESSION
  /MISSING LISTWISE
  /STATISTICS COEFF OUTS CI(95) R ANOVA
  /CRITERIA=PIN(.05) POUT(.10)
  /NOORIGIN
  /DEPENDENT HBA1C_VAL
  /METHOD=ENTER
SEX
AGE
CHFDFL
HTD1YN
DIAMFL
HYPURNYN
LMDYN
CVACYN
TEDYN
GASDYN
```

```
SLVDYN
MGTYN
BLDFL
ALZHYN
AFTHSPE1
ARTD1
ARTD2
APLD
PRPI
PGPI
AFTYP_dammy1
AFTYP_dammy2
AFTYP_dammy3
AFTYP_dammy4
  /SAVE PRED.

REGRESSION
  /MISSING LISTWISE
  /STATISTICS COEFF OUTS CI(95) R ANOVA
  /CRITERIA=PIN(.05) POUT(.10)
  /NOORIGIN
  /DEPENDENT TCMCNT_WA_CNT
  /METHOD=ENTER
SEX
AGE
CHFDFL
HTD1YN
DIAMFL
HYPURNYN
LMDYN
CVACYN
TEDYN
GASDYN
SLVDYN
MGTYN
BLDFL
```

ALZHYN
AFTHSPE1
ARTD1
ARTD2
APLD
PRPI
PGPI
AFTYP_dammy1
AFTYP_dammy2
AFTYP_dammy3
AFTYP_dammy4
  /SAVE PRED.

* By "SAVE PRED" command, a predicted value for each continuous value was yielded to all data. The names of the new parameters (predicted value) are listed below.
PRED_BMI_BL: predicted value of BMI_BL
PRED_SBP_BL: predicted value of SBP_BL
PRED_CRC: predicted value of CRC
PRED_HEMO_VAL: predicted value of HEMO_VAL
PRED_HBA1C_VAL: predicted value of HBA1C_VAL
PRED_TCMCNT_WA_CNT: predicted value of TCMCNT_WA_CNT
