# Supplementary material for: Clinical phenotypes of older adults with non-valvular atrial fibrillation not treated with oral anticoagulants by hierarchical cluster analysis in the ANAFIE Registry
Source: PLoS One. 2023 Feb 8;18(2):e0280753. doi: 10.1371/journal.pone.0280753 (PMC9907799; doi:10.1371/journal.pone.0280753)
Supplement: S4 File — (PDF) [file pone.0280753.s005.pdf]

*Description in manuscript:

For the proportion of categorical variables (no missing data, except for patients with a fall within 1 year before enrollment [missing: 11.3%]), predictive probabilities were calculated by multivariate logistic regression analysis using all categorical variables (other than the predicted variable) and age.

* Code to prepare the predicted categorical variables

```
COMPUTE FALYN_CAT = FALYN.
EXECUTE.

IF(FALYN = 9)    ADYN_NO = ''.
EXECUTE.



USE ALL.
COMPUTE filter_$=(AD_SELECTED = 1 & ADYN_NO =1).
VARIABLE LABELS filter_$ 'AD_SELECTED = 1 & ADYN_NO =1 (FILTER)'.
VALUE LABELS filter_$ 0 'Not Selected' 1 'Selected'.
FORMATS filter_$ (f1.0).
FILTER BY filter_$.
EXECUTE.

LOGISTIC REGRESSION VARIABLES CHFDFL
  /METHOD=ENTER
SEX
AGE
HTD1YN
DIAMFL
HYPURNYN
LMDYN
CVACYN
TEDYN
```

```
GASDYN
SLVDYN
MGTYN
BLDFL
ALZHYN
AFTHSPE1
ARTD1
ARTD2
APLD
PRPI
PGPI
AFTYP
  /CONTRAST (AFTYP)=Indicator(1)
  /SAVE=PRED
  /PRINT=CI(95)
  /CRITERIA=PIN(0.05) POUT(0.10) ITERATE(20) CUT(0.5).

LOGISTIC REGRESSION VARIABLES HTD1YN
  /METHOD=ENTER
SEX
AGE
CHFDFL
DIAMFL
HYPURNYN
LMDYN
CVACYN
TEDYN
GASDYN
SLVDYN
MGTYN
BLDFL
ALZHYN
AFTHSPE1
ARTD1
ARTD2
APLD
```

```
PRPI
PGPI
AFTYP
   /CONTRAST (AFTYP)=Indicator(1)
   /SAVE=PRED
   /PRINT=CI(95)
   /CRITERIA=PIN(0.05) POUT(0.10) ITERATE(20) CUT(0.5).

LOGISTIC REGRESSION VARIABLES DIAMFL
   /METHOD=ENTER
SEX
AGE
CHFDFL
HTD1YN
HYPURNYN
LMDYN
CVACYN
TEDYN
GASDYN
SLVDYN
MGTYN
BLDFL
ALZHYN
AFTHSPE1
ARTD1
ARTD2
APLD
PRPI
PGPI
AFTYP
   /CONTRAST (AFTYP)=Indicator(1)
   /SAVE=PRED
   /PRINT=CI(95)
   /CRITERIA=PIN(0.05) POUT(0.10) ITERATE(20) CUT(0.5).

LOGISTIC REGRESSION VARIABLES HYPURNYN
```

```
   /METHOD=ENTER
SEX
AGE
CHFDFL
HTD1YN
DIAMFL
LMDYN
CVACYN
TEDYN
GASDYN
SLVDYN
MGTYN
BLDFL
ALZHYN
AFTHSPE1
ARTD1
ARTD2
APLD
PRPI
PGPI
AFTYP
   /CONTRAST (AFTYP)=Indicator(1)
   /SAVE=PRED
   /PRINT=CI(95)
   /CRITERIA=PIN(0.05) POUT(0.10) ITERATE(20) CUT(0.5).

LOGISTIC REGRESSION VARIABLES LMDYN
   /METHOD=ENTER
SEX
AGE
CHFDFL
HTD1YN
DIAMFL
HYPURNYN
CVACYN
TEDYN
```

```
GASDYN
SLVDYN
MGTYN
BLDFL
ALZHYN
AFTHSPE1
ARTD1
ARTD2
APLD
PRPI
PGPI
AFTYP
  /CONTRAST (AFTYP)=Indicator(1)
  /SAVE=PRED
  /PRINT=CI(95)
  /CRITERIA=PIN(0.05) POUT(0.10) ITERATE(20) CUT(0.5).

LOGISTIC REGRESSION VARIABLES CVACYN
  /METHOD=ENTER
SEX
AGE
CHFDFL
HTD1YN
DIAMFL
HYPURNYN
LMDYN
TEDYN
GASDYN
SLVDYN
MGTYN
BLDFL
ALZHYN
AFTHSPE1
ARTD1
ARTD2
APLD
```

```
PRPI
PGPI
AFTYP
  /CONTRAST (AFTYP)=Indicator(1)
  /SAVE=PRED
  /PRINT=CI(95)
  /CRITERIA=PIN(0.05) POUT(0.10) ITERATE(20) CUT(0.5).

LOGISTIC REGRESSION VARIABLES TEDYN
  /METHOD=ENTER
SEX
AGE
CHFDFL
HTD1YN
DIAMFL
HYPURNYN
LMDYN
CVACYN
GASDYN
SLVDYN
MGTYN
BLDFL
ALZHYN
AFTHSPE1
ARTD1
ARTD2
APLD
PRPI
PGPI
AFTYP
  /CONTRAST (AFTYP)=Indicator(1)
  /SAVE=PRED
  /PRINT=CI(95)
  /CRITERIA=PIN(0.05) POUT(0.10) ITERATE(20) CUT(0.5).

LOGISTIC REGRESSION VARIABLES GASDYN
```

```
  /METHOD=ENTER
SEX
AGE
CHFDFL
HTD1YN
DIAMFL
HYPURNYN
LMDYN
CVACYN
TEDYN
SLVDYN
MGTYN
BLDFL
ALZHYN
AFTHSPE1
ARTD1
ARTD2
APLD
PRPI
PGPI
AFTYP
  /CONTRAST (AFTYP)=Indicator(1)
  /SAVE=PRED
  /PRINT=CI(95)
  /CRITERIA=PIN(0.05) POUT(0.10) ITERATE(20) CUT(0.5).

LOGISTIC REGRESSION VARIABLES SLVDYN
  /METHOD=ENTER
SEX
AGE
CHFDFL
HTD1YN
DIAMFL
HYPURNYN
LMDYN
CVACYN
```

```
TEDYN
GASDYN
MGTYN
BLDFL
ALZHYN
AFTHSPE1
ARTD1
ARTD2
APLD
PRPI
PGPI
AFTYP
  /CONTRAST (AFTYP)=Indicator(1)
  /SAVE=PRED
  /PRINT=CI(95)
  /CRITERIA=PIN(0.05) POUT(0.10) ITERATE(20) CUT(0.5).

LOGISTIC REGRESSION VARIABLES MGTYN
  /METHOD=ENTER
SEX
AGE
CHFDFL
HTD1YN
DIAMFL
HYPURNYN
LMDYN
CVACYN
TEDYN
GASDYN
SLVDYN
BLDFL
ALZHYN
AFTHSPE1
ARTD1
ARTD2
APLD
```

```
PRPI
PGPI
AFTYP
  /CONTRAST (AFTYP)=Indicator(1)
  /SAVE=PRED
  /PRINT=CI(95)
  /CRITERIA=PIN(0.05) POUT(0.10) ITERATE(20) CUT(0.5).

LOGISTIC REGRESSION VARIABLES BLDFL
  /METHOD=ENTER
SEX
AGE
CHFDFL
HTD1YN
DIAMFL
HYPURNYN
LMDYN
CVACYN
TEDYN
GASDYN
SLVDYN
MGTYN
ALZHYN
AFTHSPE1
ARTD1
ARTD2
APLD
PRPI
PGPI
AFTYP
  /CONTRAST (AFTYP)=Indicator(1)
  /SAVE=PRED
  /PRINT=CI(95)
  /CRITERIA=PIN(0.05) POUT(0.10) ITERATE(20) CUT(0.5).

LOGISTIC REGRESSION VARIABLES ALZHYN
```

```
   /METHOD=ENTER
SEX
AGE
CHFDFL
HTD1YN
DIAMFL
HYPURNYN
LMDYN
CVACYN
TEDYN
GASDYN
SLVDYN
MGTYN
BLDFL
AFTHSPE1
ARTD1
ARTD2
APLD
PRPI
PGPI
AFTYP
   /CONTRAST (AFTYP)=Indicator(1)
   /SAVE=PRED
   /PRINT=CI(95)
   /CRITERIA=PIN(0.05) POUT(0.10) ITERATE(20) CUT(0.5).

LOGISTIC REGRESSION VARIABLES AFTHSPE1
   /METHOD=ENTER
SEX
AGE
CHFDFL
HTD1YN
DIAMFL
HYPURNYN
LMDYN
CVACYN
```

```
TEDYN
GASDYN
SLVDYN
MGTYN
BLDFL
ALZHYN
ARTD1
ARTD2
APLD
PRPI
PGPI
AFTYP
  /CONTRAST (AFTYP)=Indicator(1)
  /SAVE=PRED
  /PRINT=CI(95)
  /CRITERIA=PIN(0.05) POUT(0.10) ITERATE(20) CUT(0.5).

LOGISTIC REGRESSION VARIABLES ARTD1
  /METHOD=ENTER
SEX
AGE
CHFDFL
HTD1YN
DIAMFL
HYPURNYN
LMDYN
CVACYN
TEDYN
GASDYN
SLVDYN
MGTYN
BLDFL
ALZHYN
AFTHSPE1
ARTD2
APLD
```

```
PRPI
PGPI
AFTYP
  /CONTRAST (AFTYP)=Indicator(1)
  /SAVE=PRED
  /PRINT=CI(95)
  /CRITERIA=PIN(0.05) POUT(0.10) ITERATE(20) CUT(0.5).

LOGISTIC REGRESSION VARIABLES ARTD2
  /METHOD=ENTER
SEX
AGE
CHFDFL
HTD1YN
DIAMFL
HYPURNYN
LMDYN
CVACYN
TEDYN
GASDYN
SLVDYN
MGTYN
BLDFL
ALZHYN
AFTHSPE1
ARTD1
APLD
PRPI
PGPI
AFTYP
  /CONTRAST (AFTYP)=Indicator(1)
  /SAVE=PRED
  /PRINT=CI(95)
  /CRITERIA=PIN(0.05) POUT(0.10) ITERATE(20) CUT(0.5).

LOGISTIC REGRESSION VARIABLES APLD
```

```
  /METHOD=ENTER
SEX
AGE
CHFDFL
HTD1YN
DIAMFL
HYPURNYN
LMDYN
CVACYN
TEDYN
GASDYN
SLVDYN
MGTYN
BLDFL
ALZHYN
AFTHSPE1
ARTD1
ARTD2
PRPI
PGPI
AFTYP
  /CONTRAST (AFTYP)=Indicator(1)
  /SAVE=PRED
  /PRINT=CI(95)
  /CRITERIA=PIN(0.05) POUT(0.10) ITERATE(20) CUT(0.5).

LOGISTIC REGRESSION VARIABLES PRPI
  /METHOD=ENTER
SEX
AGE
CHFDFL
HTD1YN
DIAMFL
HYPURNYN
LMDYN
CVACYN
```

```
TEDYN
GASDYN
SLVDYN
MGTYN
BLDFL
ALZHYN
AFTHSPE1
ARTD1
ARTD2
APLD
PGPI
AFTYP
  /CONTRAST (AFTYP)=Indicator(1)
  /SAVE=PRED
  /PRINT=CI(95)
  /CRITERIA=PIN(0.05) POUT(0.10) ITERATE(20) CUT(0.5).

LOGISTIC REGRESSION VARIABLES PGPI
  /METHOD=ENTER
SEX
AGE
CHFDFL
HTD1YN
DIAMFL
HYPURNYN
LMDYN
CVACYN
TEDYN
GASDYN
SLVDYN
MGTYN
BLDFL
ALZHYN
AFTHSPE1
ARTD1
ARTD2
```

```
APLD
PRPI
AFTYP
   /CONTRAST (AFTYP)=Indicator(1)
   /SAVE=PRED
   /PRINT=CI(95)
   /CRITERIA=PIN(0.05) POUT(0.10) ITERATE(20) CUT(0.5).

LOGISTIC REGRESSION VARIABLES FALYN_CAT
   /METHOD=ENTER
SEX
AGE
CHFDFL
HTD1YN
DIAMFL
HYPURNYN
LMDYN
CVACYN
TEDYN
GASDYN
SLVDYN
MGTYN
BLDFL
ALZHYN
AFTHSPE1
ARTD1
ARTD2
APLD
PRPI
PGPI
AFTYP
   /CONTRAST (AFTYP)=Indicator(1)
   /SAVE=PRED
   /PRINT=CI(95)
   /CRITERIA=PIN(0.05) POUT(0.10) ITERATE(20) CUT(0.5).
```

* By "SAVE=PRED" command, a predicted value for each categorical value was yielded to all data. The names of the new parameters (predicted value) are listed below.

PRED_CHFDFL: predicted value of CHFDFL

PRED_HTD1YN: predicted value of HTD1YN

PRED_DIAMFL: predicted value of DIAMFL

PRED_HYPURNYN: predicted value of HYPURNYN

PRED_LMDYN: predicted value of LMDYN

PRED_CVACYN: predicted value of CVACYN

PRED_TEDYN: predicted value of TEDYN

PRED_GASDYN: predicted value of GASDYN

PRED_SLVDYN: predicted value of SLVDYN

PRED_MGTYN: predicted value of MGTYN

PRED_BLDFL: predicted value of BLDFL

PRED_ALZHYN: predicted value of ALZHYN

PRED_AFTHSPE1: predicted value of AFTHSPE1

PRED_ARTD1: predicted value of ARTD1

PRED_ARTD2: predicted value of ARTD2

PRED_APLD: predicted value of APLD

PRED_PRPI: predicted value of PRPI

PRED_PGPI: predicted value of PGPI

PRED_FALYN_CAT: predicted value of FALYN_CAT
