# Supplementary material for: Clinical phenotypes of older adults with non-valvular atrial fibrillation not treated with oral anticoagulants by hierarchical cluster analysis in the ANAFIE Registry
Source: PLoS One. 2023 Feb 8;18(2):e0280753. doi: 10.1371/journal.pone.0280753 (PMC9907799; doi:10.1371/journal.pone.0280753)
Supplement: S5 File — (PDF) [file pone.0280753.s006.pdf]

*Description of manuscript
2) Hierarchical cluster analysis
A hierarchical cluster analysis was performed using Ward's linkage hierarchical algorithm on predicted values for continuous variables and predictive probabilities for categorical variables [11].

* Code to perform the cluster analysis

```
USE ALL.
COMPUTE filter_$=(AD_SELECTED = 1 & ADYN_NO =1).
VARIABLE LABELS filter_$ 'AD_SELECTED = 1 & ADYN_NO =1 (FILTER)'.
VALUE LABELS filter_$ 0 'Not Selected' 1 'Selected'.
FORMATS filter_$ (f1.0).
FILTER BY filter_$.
EXECUTE.

DATASET DECLARE D0.5695737675031739.
PROXIMITIES
AGE
SEX
PRED_BMI_BL
PRED_SBP_BL
PRED_CRC
PRED_HEMO_VAL
PRED_HBA1C_VAL
PRED_TCMCNT_WA_CNT
PRED_CHFDFL
PRED_HTD1YN
PRED_DIAMFL
PRED_HYPURNYN
PRED_LMDYN
PRED_CVACYN
PRED_TEDYN
PRED_GASDYN
```

```
PRED_SLVDYN
PRED_MGTYN
PRED_BLDFL
PRED_ALZHYN
PRED_AFTHSPE1
PRED_ARTD1
PRED_ARTD2
PRED_APLD
PRED_PRPI
PRED_PGPI
PRED_FALYN_CAT
AFTYP
  /MATRIX OUT(D0.5695737675031739)
  /VIEW=CASE
  /MEASURE=SEUCLID
  /PRINT NONE
  /STANDARDIZE=VARIABLE Z.
CLUSTER
  /MATRIX IN(D0.5695737675031739)
  /METHOD WARD
  /PRINT SCHEDULE CLUSTER(2,4)
  /PLOT DENDROGRAM VICICLE
  /SAVE CLUSTER(2,4).
Dataset Close D0.5695737675031739.
```

* By "SAVE CLUSTER(2,4)" command, three parameters were yielded, named cluster2, cluseter3, and cluster4, with which all data were assigned to two clusters, three clusters, and four clusters, respectively. Since we employed the two clusters, the parameter of "cluster2" was used for the further analyses.
