# Supplementary material for: Clinical phenotypes of older adults with non-valvular atrial fibrillation not treated with oral anticoagulants by hierarchical cluster analysis in the ANAFIE Registry
Source: PLoS One. 2023 Feb 8;18(2):e0280753. doi: 10.1371/journal.pone.0280753 (PMC9907799; doi:10.1371/journal.pone.0280753)
Supplement: S6 File — (PDF) [file pone.0280753.s007.pdf]

```
* Code to analyze the patient characteristics


COMPUTE CCR50LT = 0.
EXECUTE.

IF(CCR < 50) CCR50LT = 1.
EXECUTE.

COMPUTE LBMI = 0.
EXECUTE.

IF(BMI_BL < 18.5) LBMI = 1.
EXECUTE.

COMPUTE PEAF = 0.
EXECUTE.

IF(AFTYP >= 2) PEAF = 1.
EXECUTE.

COMPUTE HELTES = AGE75FL + AGEC2 + HYPTFL + LBMI + PEAF + TIATEFL.
EXECUTE.




USE ALL.
COMPUTE filter_$=(AD_SELECTED = 1 & ADYN_NO =1).
VARIABLE LABELS filter_$ 'AD_SELECTED = 1 & ADYN_NO =1 (FILTER)'.
VALUE LABELS filter_$ 0 'Not Selected' 1 'Selected'.
FORMATS filter_$ (f1.0).
FILTER BY filter_$.
EXECUTE.

SPLIT FILE OFF.
```

```
CROSSTABS
  /TABLES=
SEX
AGEC2
BLDFL
AFTHSPE
AFTHSPE1
AFTHSPE2
AFTHSPE3
AFTHSPE4
AFTHSPE5
CHFDFL
HTD1YN
DIAMFL
HYPURNYN
KDDS1YN
LMDYN
CVACYN
TEDYN
GASDYN
SLVDYN
MGTYN
ALZHYN
ARTD1
ARTD2
APLD
PRPI
PGPI
CCR50LT
HYPURNYN
TEDYN
  BY cluster2
  /FORMAT=AVALUE TABLES
  /STATISTICS=CHISQ
  /CELLS=COUNT ROW COLUMN
```

```
   /COUNT ROUND CELL.

CROSSTABS
   /TABLES=
AFTYP
FALYN_CAT
CHADS2
CHA2DS2V
HASBLED
AGEC
   BY cluster2
   /FORMAT=AVALUE TABLES
   /STATISTICS=CHISQ
   /CELLS=COUNT ROW COLUMN
   /COUNT ROUND CELL.

DESCRIPTIVES VARIABLES=
AGE
BMI_BL
SBP_BL
DBP_BL
CRC
HBA1C_VAL
CHADS2
CHA2DS2V
HELTES
HASBLED
TCMCNT_WA
   /STATISTICS=MEAN STDDEV MIN MAX.


T-TEST GROUPS=cluster2(1 2)
   /MISSING=ANALYSIS
   /VARIABLES=
AGE
BMI_BL
```

```
SBP_BL
DBP_BL
CRC
HBA1C_VAL
CHADS2
CHA2DS2V
HELTES
HASBLEDTCMCNT_WA
   /ES DISPLAY(TRUE)
   /CRITERIA=CI(.95).

CROSSTABS
   /TABLES=
CRCC
   BY cluster2
   /FORMAT=AVALUE TABLES
   /STATISTICS=CHISQ
   /CELLS=COUNT ROW COLUMN
   /COUNT ROUND CELL.
```
