# Supplementary material for: Clinical phenotypes of older adults with non-valvular atrial fibrillation not treated with oral anticoagulants by hierarchical cluster analysis in the ANAFIE Registry
Source: PLoS One. 2023 Feb 8;18(2):e0280753. doi: 10.1371/journal.pone.0280753 (PMC9907799; doi:10.1371/journal.pone.0280753)
Supplement: S7 File — (PDF) [file pone.0280753.s008.pdf]

```
* Code to analyze the event data

COMPUTE MACNE = 0.
EXECUTE.

IF(ADTTE118 + ADTTE101 + ADTTE114 >0 ) MACNE = 1.
EXECUTE.

COMPUTE MACNE_TIME_M = MIN(ADTTE118_TIME_M, ADTTE101_TIME_M,
ADTTE114_TIME_M)
EXECUTE.



USE ALL.
COMPUTE filter_$=(AD_SELECTED = 1 & ADYN_NO =1).
VARIABLE LABELS filter_$ 'AD_SELECTED = 1 & ADYN_NO =1 (FILTER)'.
VALUE LABELS filter_$ 0 'Not Selected' 1 'Selected'.
FORMATS filter_$ (f1.0).
FILTER BY filter_$.
EXECUTE.

SPLIT FILE OFF.

KM ADTTE119_TIME_M BY cluster2
  /STATUS=ADTTE119(1)
  /PRINT TABLE MEAN
  /PLOT SURVIVAL OMS
  /TEST LOGRANK
  /COMPARE OVERALL POOLED.

KM MACNE_TIME_M BY cluster2
  /STATUS=MACNE(1)
  /PRINT TABLE MEAN
  /PLOT SURVIVAL OMS
  /TEST LOGRANK
```

```
  /COMPARE OVERALL POOLED.

KM ADTTE101_TIME_M BY cluster2
  /STATUS=ADTTE101(1)
  /PRINT TABLE MEAN
  /PLOT SURVIVAL OMS
  /TEST LOGRANK
  /COMPARE OVERALL POOLED.

KM ADTTE106_TIME_M BY cluster2
  /STATUS=ADTTE106(1)
  /PRINT TABLE MEAN
  /PLOT SURVIVAL OMS
  /TEST LOGRANK
  /COMPARE OVERALL POOLED.

KM ADTTE110_TIME_M BY cluster2
  /STATUS=ADTTE110(1)
  /PRINT TABLE MEAN
  /PLOT SURVIVAL OMS
  /TEST LOGRANK
  /COMPARE OVERALL POOLED.

KM ADTTE109_TIME_M BY cluster2
  /STATUS=ADTTE109(1)
  /PRINT TABLE MEAN
  /PLOT SURVIVAL OMS
  /TEST LOGRANK
  /COMPARE OVERALL POOLED.

KM ADTTE115_TIME_M BY cluster2
  /STATUS=ADTTE115(1)
  /PRINT TABLE MEAN
  /PLOT SURVIVAL OMS
  /TEST LOGRANK
  /COMPARE OVERALL POOLED.
```

```
DESCRIPTIVES VARIABLES=
ADTTE119
ADTTE119_TIME_M
ADTTE101
ADTTE101_TIME_M
ADTTE106
ADTTE106_TIME_M
ADTTE110
ADTTE110_TIME_M
ADTTE109
ADTTE109_TIME_M
ADTTE115
ADTTE115_TIME_M
MACNE
MACNE_TIME_M
  /STATISTICS=MEAN SUM STDDEV MIN MAX.

SORT CASES  BY cluster2 (A).
SPLIT FILE SEPARATE BY cluster2.

DESCRIPTIVES VARIABLES=
ADTTE119
ADTTE119_TIME_M
ADTTE101
ADTTE101_TIME_M
ADTTE106
ADTTE106_TIME_M
ADTTE110
ADTTE110_TIME_M
ADTTE109
ADTTE109_TIME_M
ADTTE115
ADTTE115_TIME_M
MACNE
MACNE_TIME_M
```

/STATISTICS=MEAN SUM STDDEV MIN MAX.
